# A detection dog for obstructive sleep apnea: could it work in diagnostics?

**DOI:** 10.1007/s11325-020-02113-1

**Published:** 2020-05-29

**Authors:** Jenni Vaarno, Jyri Myller, Adel Bachour, Heli Koskinen, Leif Bäck, Tuomas Klockars, Anni Koskinen

**Affiliations:** 1 Department of Otorhinolaryngology – Head and Neck Surgery, Helsinki University Hospital and University of Helsinki, PO Box 263, HUS, 00029 Helsinki, Finland; 2 Department of Otorhinolaryngology, Päijät-Häme Central Hospital, Lahti, Finland; 3 Sleep Unit, Heart and Lung Center, Helsinki University Hospital and University of Helsinki, Helsinki, Finland

**Keywords:** Obstructive sleep apnea, OSA, Detection dog, Screening, Diagnostics

## Abstract

**Purpose:**

We have previously demonstrated that dogs can be trained to distinguish the urine of patients with obstructive sleep apnea (OSA) from that of healthy controls based on olfaction. Encouraged by these promising results, we wanted to investigate if a detection dog could work as a screening tool for OSA. The objective of this study was to prospectively assess the dogs’ ability to identify sleep apnea in patients with OSA suspicion.

**Methods:**

Urine samples were collected from 50 patients suspected of having OSA. The urine sample was classified as positive for OSA when the patient had a respiratory event index of 5/h or more. The accuracy of two trained dogs in identifying OSA was tested in a prospective blinded setting.

**Results:**

Both of the dogs correctly detected approximately half of the positive and negative samples. There were no statistically significant differences in the dogs’ ability to recognize more severe cases of OSA, as compared to milder cases.

**Conclusion:**

According to our study, dogs cannot be used to screen for OSA in clinical settings, most likely due to the heterogenic nature of OSA.

## Introduction

Polysomnography (PSG) is the standard method for diagnosing obstructive sleep apnea (OSA). However, there is an urgent need to develop new approaches to diagnose and screen for OSA due to its high prevalence and the relatively limited access to PSG. Meanwhile, there exist substantial health care and social costs of undiagnosed OSA [1]. An ideal screening tool would be accurate, affordable, easy to use, and highly accessible with no side effects to the patient [2].

Within this context, there may be a role for detection dogs. Dogs possess excellent proficiency at detecting a wide range of scents. Their olfactory sensitivity can be up to 100,000 times better than that of humans. Dogs have been successfully trained to detect different cancers [3–5] and infectious diseases [6, 7].

Medical detection dogs could represent an economical and robust option in screening for OSA. We have previously demonstrated that dogs can be trained to distinguish OSA urine from healthy control samples based on olfaction [8]. Two of our three dogs correctly detected two-thirds of OSA patient samples.

Our aim was to assess the dogs’ ability to identify sleep apnea in a prospective study targeting patients with suspected OSA.

## Materials and methods

### Dogs

The dogs used in this study were a German Spitz Mittel (Dog 1; female, 4 years old) and a Labrador Retriever (Dog 2; female, 4 years old). Both the dogs and trainers had previous experience with olfactory-based detection, and they had previously been trained and tested for OSA detection in urine samples [8].

### Training

The initial training period was 1–2 months. The training was based exclusively on operant conditioning with positive reinforcement. The correct response for the OSA sample was either sitting in front of the sample or standing still and pointing the nose at the sample for a minimum of 5 s. The correct response for negative samples was to ignore the sample.

### Patients and urine samples

Urine samples were collected from 50 patients who were referred to the Department of Otorhinolaryngology in Päijät-Häme Central Hospital on suspicion of OSA. The patient was suspected of having OSA if he/she presented with the following symptoms: snoring and/or witnessed apneas and daytime tiredness. All patients underwent a cardio-respiratory sleep study. Urine samples were fractioned to small microcentrifuge tubes and frozen at − 18 °C.

### Sleep studies

All patients underwent a cardiorespiratory overnight sleep study using a portable monitor (Nox T3, Nox Medical, Iceland). Scoring was performed according to the AASM recommendations [9]. Sleep apnea was considered present when the respiratory event index (REI) was ≥ 5/h. Otherwise, the urine samples were considered negative for sleep apnea.

### The test

The trainer was given a total of 100 samples, two samples from each of the 50 patients. Dogs were presented two to four samples daily, one sample at a time, and they had to identify whether or not it was the target odour (OSA). All 50 patient samples were tested once (1st try) and later, the second samples were tested (2nd try) so that each patient was tested twice. The trainer was blinded, receiving a random number assigned to the sample in order to avoid any possibility of signalling between the trainer and the dog.

### Statistics

The number of studied subjects was calculated according to Casagrande and Pike [10]. According to these researchers, a non-trained dog has a 50% chance of giving a right answer. If we accept an α risk at 0.05 and a β risk at 0.05, we needed a minimum of 42 subjects for our study. A chi-square test provided a comparison of the positive answers for dog 1 and dog 2. A *p* value < 0.05 was considered to be statistically significant. Analyses were performed using IBM SPSS Statistics for Windows (version 25.0).

## Results

The 50 patients represented the following OSA categories: normal (REI < 5; *n* = 7; 14%), mild (5 ≤ REI < 15; *n* = 16; 32%), moderate (15 ≤ REI <30; *n* = 10; 20%), and severe (REI ≥ 30; *n* = 17; 34%). Table 1 illustrates these patient characteristics.

**Table 1:** Patient characteristics in OSA categories

	No OSA	Mild OSA	Moderate OSA	Severe OSA
Number of patients	7	16	10	17
Women, *n* (%)	1 (14)	4 (25)	3 (30)	3 (18)
Smoker, *n* (%)	0 (0)	0 (0)	2 (20)	4 (24)
Age, mean (SD)	41 (14)	48 (12)	60 (8)	56 (14)
BMI, mean (SD)	28 (4)	28 (2)	31 (7)	33 (6)
REI, mean (SD)	2 (3)	9 (3)	24 (4)	51 (21)

No OSA = REI < 5, Mild = 5 ≤ REI <15, Moderate = 15 ≤ REI < 30, Severe = REI ≤ 30

*REI* respiratory event index, *BMI* body mass index, *SD* standard deviation, *n* number

**Fig. 1:**
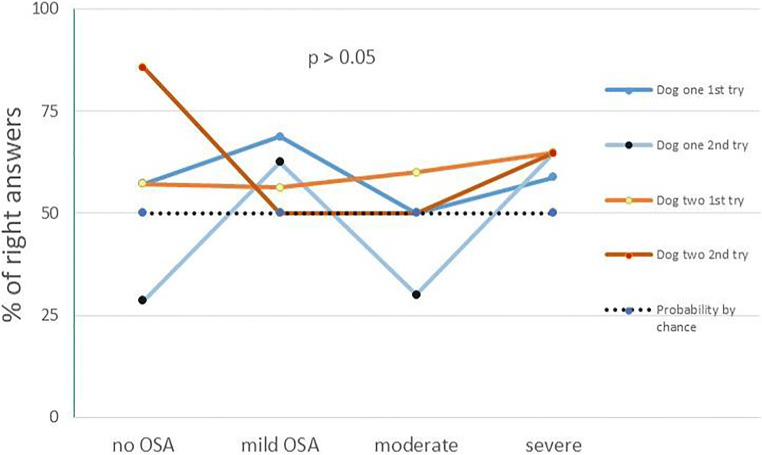
Dogs’ percentage (%) of right answers in OSA categories: No OSA = REI < 5, mild = 5 ≤ REI <15, moderate = 15 ≤ REI < 30, severe = REI ≤ 30. REI, respiratory event index

Dog 1 was able to give the right answer in 53% of cases [χ^2^ (3, *N* = 100) = 7.353, (*p* = 0.061)] and dog 2 in 52% of cases [χ^2^ (3, *N* = 100) = 3982, (*p* = 0.263)]. Figure 1 indicates the percentages (%) of right answers in terms of recognizing OSA according to the severity classification categories.

## Discussion

The chief finding of this study was that the two trained dogs were unable to distinguish between urine samples of patients with OSA from those of control subjects who did not have OSA. This result was surprising since in our previous study, the same dogs correctly detected two-thirds of patients with OSA based on olfaction, with impressive values of statistical significance (*p* < 0.000003) [8].

Our findings were also disappointing in that the proficiency of dogs in detecting a wide range of scents has shown promise for medical screening purposes in other settings. As examples, dogs’ specificity and sensitivity for detecting and discriminating cancer have been reported to be above 90% [11–13]. Promising results have been obtained for infectious diseases, such as *Clostridium difficile* infections [6, 14] and urinary tract infections [7, 15]. Unfortunately, despite the encouraging results of our first study, it seems that dogs cannot be used to screen patients with OSA. Both of our dogs only detected about half of the positive and negative samples.

There are several possible reasons for this failure. First, OSA patients are known to have changes in their urine metabolites [16, 17]. In our first study, we compared samples from OSA patients with samples from healthy individuals [8]. In this second study, urine samples came from individuals with suspected OSA. It is possible that the dogs identified abnormal scents related to OSA rather than scents specific to OSA.

Second, OSA is a heterogeneous disease with several phenotypes reported [18]. This heterogeneity also may influence the metabolic processes and volatile compound profile of the patient’s urine regardless of the severity of the disease. If so, dogs may recognize only a fraction of OSA patients belonging to certain phenotypes. This hypothesis is supported by inconsistent findings in studies examining possible biomarkers for OSA [16].

Finally, the testing procedure may have influenced the results. In the first study, dogs were presented with four samples, one of which was positive for OSA [8]. In this study, one sample was presented at a time, and the dog had to identify whether or not it was the target odour. Instead of asking “Which one?”, the dog was challenged by asking “Is it?”. Such identification tasks tend to be more challenging than discrimination by comparison.

Contrary to our promising preliminary findings, the results of this study indicate that dogs cannot be used to screen for OSA in clinical settings. Detection dogs may not be a suitable screening method for heterogeneous conditions, such as OSA.
